# Effects of Vaccine Literacy, Health Beliefs, and Flu Vaccination on Perceived Physical Health Status among Under/Graduate Students

**DOI:** 10.3390/vaccines11040765

**Published:** 2023-03-30

**Authors:** En-Jung Shon, Lena Lee

**Affiliations:** 1 Department of Social Welfare, Duksung Women’s University, Seoul 01369, Republic of Korea; 2 Department of Teaching, Curriculum, and Educational Inquiry, Miami University, Oxford, OH 45056, USA; leel@miamioh.edu

**Keywords:** vaccine literacy, health beliefs, vaccination, perceived health status

## Abstract

Physicians highlight that receiving the flu vaccine is critical, especially during the COVID-19 pandemic period. Younger groups’ flu vaccination coverage is very low, and this tendency is potentially related to a lower level of vaccine literacy and perceptions toward vaccination. This study investigated the relationship between flu vaccine literacy, health beliefs, and flu vaccinations (benefit, barrier, severity, and susceptibility) and their impact on perceived health status controlling for socioeconomic factors. It used the Health Belief Model andHealth Literacy Skills Framework with under/graduate students (*N* = 382) in Ohio, U.S. Path analyses were performed to examine the causal process using SPSS and Amos 23.0. Indicators of CFI, RMSEA, SRMR, and the chi-square/df of the path models were good–acceptable. Vaccine literacy directly impacted on health beliefs and vaccination. Susceptibility belief directly influenced perceived health status. The mediation effects of health beliefs (benefit, barrier) between vaccine literacy and vaccination were confirmed. The study highlights the need for healthcare providers and governments to work together to improve flu vaccine literacy and reduce negative perceptions toward vaccination among younger populations. Educational programs and official communication channels can be used to address concerns and provide accurate information about vaccines to increase flu vaccination rates and protect public health.

## 1. Introduction

It is widely acknowledged that flu is a prevalent and sometimes deadly virus that claims thousands of American lives each year. Physicians emphasize the importance of flu vaccinations, particularly during the ongoing COVID-19 pandemic. The Center for Disease Control and Prevention (CDC) mentioned that with the winter and flu season approaching, there is a chance that the illnesses and fatalities associated with flu could further burden the healthcare systems that are already strained due to the continuing COVID-19 pandemic. Healthcare providers said that it is essential to keep flu under control so that hospitals can focus on caring for individuals with COVID-19 [1]. Marsa (2021, October) in the National Geographic Society also reported that experts are continuously warning that there is a high probability of a severe flu outbreak in the upcoming months in the U.S., which emphasizes the importance of being vaccinated for both illnesses now more than ever [2]. Healthcare authorities should therefore continue to raise awareness about the need to increase flu vaccinations among the general public [3].

Flu vaccine coverage among young adults is typically lower compared to older adults [4]. In 2021–2022, the lowest level of vaccination coverage was reported among young and middle-aged adults: overall coverage was 49.4%, with 37.1% for 18–49-year-olds, 52.4% for 50–64-year-olds, and 73.9% for 65-year-olds and older [5]. Despite the importance of receiving the flu vaccine, which is one of the most effective methods for preventing flu infections and reducing hospitalizations and deaths caused by influenza [6], young and middle-aged groups continue to have low vaccination rates. The low vaccination rate among youth may be due to a lack of vaccine literacy [7,8] and the belief of having a lower susceptibility to flu and its consequences [4]. However, prior research reported that younger adults could be vulnerable to the severe effects of seasonal flu [9,10]. For instance, college or university students have been classified as high-risk groups due to their age and communal living arrangements, such as living together in dormitories, during both pandemic and non-pandemic years. Flu and its associated illnesses can cause a significant burden on the health and academic/work performance of younger adults [11,12]. Additionally, we must acknowledge that younger groups have the potential to (unintentionally) transmit illnesses to other individuals (e.g., older adults and those with weakened immune systems) in the community who are more susceptible to severe complications and hospitalizations from flu-related diseases [4]. Therefore, understanding the factors that influence young people’s vaccination decisions is crucial for preventing the spread of the virus and protecting all age groups from flu infections. This study focuses on the effects of vaccine literacy on health beliefs, vaccination behaviors, and perceived health status among younger adults.

According to Healthy People 2030 [13] (p. S259), health literacy is the level of individuals’ capability to discover, comprehend, and utilize given information and services to make informed health-related decisions and take action for themselves and others. While health literacy has been shown to improve health outcomes and reduce health disparities [3], previous research found that low health literacy is linked to negative health outcomes, including frequent hospitalization [14,15], higher mortality rates [16,17], non-compliance with medications [18], and lower vaccination rates [7,16,19].

Poor health outcomes are directly and indirectly related to health behaviors [20], and health literacy plays a crucial role in shaping perceptions and beliefs about preventive behaviors such as vaccination and health beliefs. Individuals’ health behaviors are related to their perceived risk of infection and severity of the disease and the perceived benefits and barriers [21]. Brega et al. (2021) [22] found that health literacy is linked to health beliefs, including perceived severity, benefits, and barriers. Ghorbani-Dehbalaei et al. (2021) [23] similarly pointed out that both health literacy and health beliefs—specifically, perceived susceptibility—are associated with preventive behaviors. Several studies have found that perceived benefits [24,25,26], susceptibility [25,27], and self-efficacy [26,28] are significant factors in determining the intention of younger adults to be vaccinated. As a result, it is crucial to improve the beneficial impact of vaccine literacy on vaccine beliefs and vaccination.

With regard to health outcomes, the flu vaccine may play a crucial role in not only providing immunity against infection for individuals but also protecting them from contracting illnesses. Many studies show that flu vaccination is effective in reducing flu infections, hospitalizations, and negative health outcomes [20,29,30,31]. Gross et al. (1995) [32] conducted a meta-analysis showing the efficacy of flu vaccination on health outcomes, and Demicheli et al. (2000) [33] found strong correlations between flu vaccination and both clinical and self-reported health outcomes through a systematic review.

The combination of the Health Belief Model (HBM) and the Health Literacy Skills Framework (HLSF) was applied as a theoretical frame. The HBM [34] and the HLSF [35] demonstrate the impact of health literacy on health beliefs, behavior changes, and outcomes. HLSF posits that many factors mediate the relationship between health literacy skills (such as print literacy, communication skills, and information seeking and utilization) and health behaviors or outcomes. These mediators may include perceptions, motivations, attitudes, or self-efficacy, and are tied to individual beliefs. For its part, the HBM [34] is a well-known framework representing the core health belief concepts of individuals. It includes perceived benefits (e.g., beliefs about the positive aspects of adopting a health behavior), perceived barriers (e.g., beliefs about the obstacles to performing a behavior and their negative aspects), perceived severity (e.g., beliefs about the seriousness of contracting a disease or condition, including consequences), and perceived susceptibility (e.g., beliefs about the likelihood of getting a disease or condition) [23,34]. 

**Figure 1:**
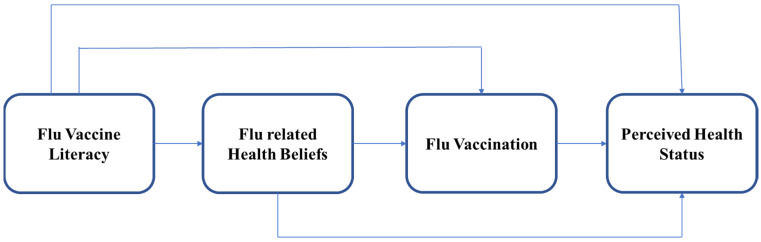
Study Design for the Current Study. Flu Vaccine literacy, Flu-related Health Beliefs, Flu Vaccination, and Perceived Health Status.

**Figure 2:**
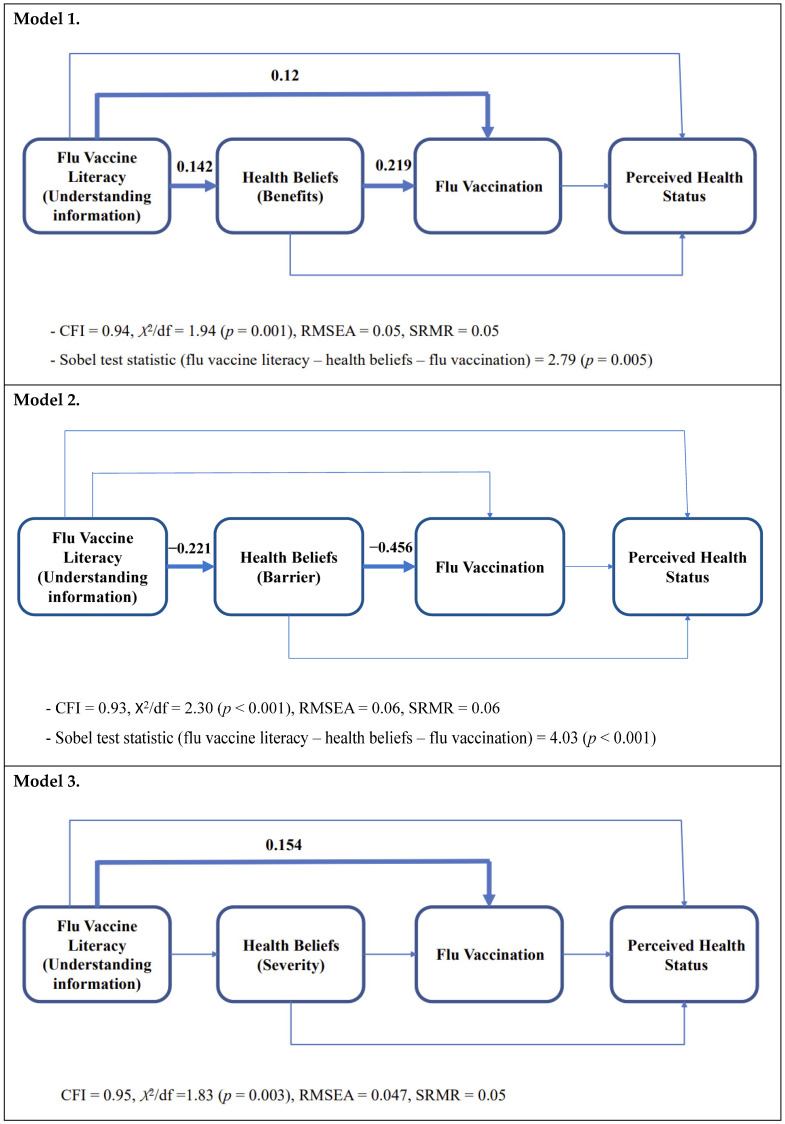

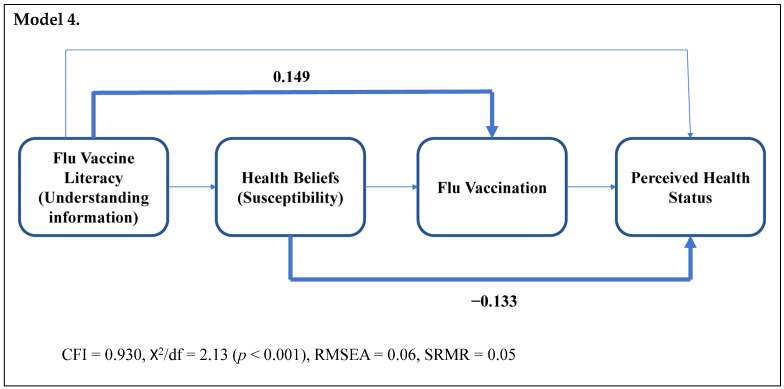
Effect of Flu Vaccine Literacy (i.e., understanding information) on Flu-related Health Beliefs, Flu Vaccination, and Perceived Physical Health Status. Note: Controlled factors: race, school year, gender, household income, and education attainment of the survey participants’ parents (mother and father); significant relationships were presented with boldlines; Sobeltest scores were presented for the models reporting the significant medication effects.

As shown in Figure 1, the present study proposed a mediation model (health literacy–health belief–flu vaccination–perceived health status), whose central hypothesis, after controlling for socioeconomic factors, is that flu vaccine literacy influences the components of health beliefs and that flu vaccination, in turn, affects perceived health status. The concept of the flu vaccine literacy and the four components of health beliefs (perceived benefits, barriers, severity, and susceptibility) were separately applied to the study design. The following specific hypotheses were proposed:

-
  **Hypothesis** **1.** *Flu vaccine literacy influences perceived benefits and flu vaccination influences perceived health status, controlling for socioeconomic factors.*
-
  **Hypothesis** **2.** *Flu vaccine literacy influences perceived barriers and flu vaccination influences perceived health status, controlling for socioeconomic factors.*
-
  **Hypothesis** **3.** *Flu vaccine literacy influences perceived severity and flu vaccination influences perceived health status, controlling for socioeconomic factors.*
-
  **Hypothesis** **4.** *Flu vaccine literacy influences perceived susceptibility and flu vaccination influences perceived health status, controlling for socioeconomic factors.*

(The above hypotheses are tested in Models 1–4 in Figure 2 in the study’s Methods and Results sections.)

## 2. Materials and Methods

### 2.1. Data Collection Procedure

The majority of student health centers in U.S. universities are providing free vaccines to their students, faculty, and staff members, which means that accessing the vaccine is not a significant barrier for this population. The current study conducted in Ohio State areas focused specifically on undergraduate and graduate students (18 years and older), who are typically younger adults. This suggests that the study aimed to gather information about the flu vaccination behaviors among a specific age group (i.e., younger adults: under/graduate students). To be specific, data collection was conducted through a convenient sampling method, targeting undergraduate/graduate students from two universities and several community churches in Ohio, U.S. The aim was to enhance the generalizability of the findings to college students in Ohio. By targeting both universities and community churches, this study could enhance the generalizability for college or university students in Ohio. Approval for the data collection was obtained from the Institutional Review Board (IRB) of the lead university.

A self-administered web-based survey was conducted from September 2019 to March 2020. After the IRB approval, participants were recruited through e-mail, obtained from the universities and community churches, and the data collection process was managed by the research team’s principal investigator (PI). A sample of student e-mails for the data collection was provided from the Institutional Research and Effectiveness of the lead universities. Appointed people from the community churches gathered e-mails of the potential survey participants and handed them to the PI of the research team. Web-based data collection procedures were also managed by the PI. Potential participants were initially asked to read the survey guideline and the consent form. Based on the agreement on the consent form, the students were automatically guided to the main questionnaires. The survey included socioeconomic characteristics (race, gender, family income, school year (1st–5th year), marital status of parents, insurance status), flu-vaccine-related literacy [36], Health Belief Model questionnaires [37,38,39], flu vaccination, and perceived health status. A total of 526 individuals participated in the survey. Out of the 526, only 382 participants were considered for analysis using the listwise method. The sample population was ethnically diverse, with 37.4% identifying as White, 15.4% as African American, 18.1% as Hispanic, and 29.1% as Asian. For gender, 73.8% were female, and 26.2% were male. The mean age of the collected group was 22.37 (*SD* = 5.97). More details of the sample characteristics are introduced in the Results section.

### 2.2. Measures

‘Perceived Health Status’ was asked, “how would you describe your physical health in general?” (response option: 1 = poor~5= excellent; quasi-interval form; observed range = 1–5; skewness score = |0.24|). For the ‘flu Vaccination’, the question of “During the past 12 months, have you had a flu vaccine?” (response option: 1 = yes, 0 = no) was asked.

For ‘the Vaccine Literacy’, the concepts of ‘understanding information (i.e., “Based on given information, do you feel sure about the best choice [vaccinated versus non-vaccinated] for you to prevent flu infection?”)’ was applied (response options: 1 = yes or 0 = no). This measure was generated from the core concept of the Ottawa Decision Support Framework [36]. Regarding the questions of ‘perceived health status’ and ‘flu vaccination behavior’, representative public data were used as benchmarks. The ‘Health and Retirement Study’, managed by the University of Michigan in the US, and the ‘California Health Interview Survey’, managed by the University of California, Los Angeles (UCLA) Center for Health Policy Research, use the same questionnaires to assess perceived health status and flu vaccination behavior. The study also measured ‘Health Beliefs’ using 16 items [37,38,39]. These items addressed various aspects of the participants’ beliefs about flu vaccination, such as opposition, side-effects, convenience, cost, and effectiveness. Details were as follows (see the Table 1):

The study also included several socioeconomic factors: race, gender, family household income, school year, and parent’s education level. Race was recoded into four categories: White, African American, Hispanic or Latino, and Asian or Pacific Islander. Gender was recorded as binary, with female = 1 and male = 0. Family household income was recoded as an open-ended question, with a range of USD 0–5,000,000, and then recoded based on skewness and kurtosis into three categories: 1 = USD 0 to USD 44,999, 2 = USD 45,000 to USD 139,999, and 3 = USD 140,000 to USD 5,000,000). The school year was recoded on a scale of 1 (1st-year undergraduate) to 6 (graduate student). Parents’ education level was recoded on a scale of 0 (no formal education) to 8 (doctorate or higher). Response option details of parents’ educational attainment were as follows: (0 = no formal education, 1 = elementary school, 2 = middle school, 3 = high school, 4 = some college, 5 = 2 years college, 6 = 4 years university, 7 = master’s degree, 8 = doctorate or higher). For rationale of selecting above socioeconomic factors, prior studies were reviewed [3,4,25,26].

### 2.3. Analyses Strategies

For data analyses, SPSS and AMOS 23.0 were used. For the complete data (out of 526 survey participants), the listwise method was applied with the final sample size, 382 [40].

Univariate frequencies, descriptive statistics, histograms, and bivariate scatter plots were used to identify outliers (>±2 SD) and evaluate variability. As for continuous measures, skewness was evaluated using guidelines [41]. Path analyses were conducted to examine the impact of vaccine literacy, health beliefs, flu vaccination, and perceived physical health. Regression imputation maximum likelihood was used as an option. The fit of the path models was evaluated using *X*^2^/df with p-value, Comparative Fit Index (CFI), Root Mean Square Error or Approximation (RMSEA), and Standardized Root Mean Square Residual (SRMR) [40,42]. After reviewing the prior studies, statistical controls were applied for socioeconomic factors including race, school year, gender, household income, and parent’s education level [43,44,45,46]. Multivariate data analyses, such as path analyses, can statistically adjust control variables and, as a result, suggest the main effects of the analyses.

## 3. Results

### 3.1. Sample Characteristics

Table 2 presents details of the sample characteristics. As mentioned earlier, there were 382 individuals in the final group, with 49% (*N* = 187) having received the flu vaccine and 51% (*N* = 195) not having received it. The average household income was USD 144,237.49, with a range of USD 0 to USD 500,000 and a standard deviation of USD 285,290.28. To be specific, 24.1% of participants had a household income of USD 0 to USD 44,999, 42.9% had an income of USD 45,000 to USD 139,999, and 33% had an income above USD 140,000. On average, participants’ parents graduated after 2 years of college (response option: 0 = no formal education~8 = doctorate or higher). The average education level of under/graduate students was third year (senior students). Among the sample characteristics presented in Table 2, race, gender, annual household income, educational attainment of undergraduate and graduate students, and educational attainment of parents were statistically controlled. Age and educational attainment were found to be highly correlated, and therefore educational attainment was selected for analysis. Concerning insurance status, the majority of the target group was insured, regardless of the type of insurance. This manuscript considered the sufficient variability of the response options for insurance status, and therefore insurance status was not included as a statistical control variable.

### 3.2. Hypothesis Testing

Based on the suggested evaluation criteria [40,42], the path models reported good model fit scores. The CFI, RMSEA, SRMR, and the chi-square/df were in the good–acceptable range.

Mediation Effect. Figure 2 shows that the models (1–4) examining the impact of flu vaccine literacy (i.e., understanding information as “Based on the given information, do you feel sure about the best choice [vaccinated vs. non-vaccinated] to prevent flu infection?”) on health beliefs, flu vaccination, and perceived health status showed good–fair model fit [40,42]. Models 1 and 2 (Figure 2) show that health beliefs mediated the relationship between flu vaccine literacy and flu vaccination. Individuals understanding information (i.e., “Based on given information, do you feel sure about the best choice [vaccinated versus non-vaccinated] for you to prevent flu infection?”) reported higher levels of positive health beliefs (i.e., benefits; standardized estimate = 0.142, *p* = 0.005); in turn, they were more likely to have had the flu vaccine than their counterparts (standardized estimate = 0.219, *p* < 0.001; Sobel test score = 2.79, *p* = 0.005). Interestingly, individuals understanding information (i.e., “Based on given information, do you feel sure about the best choice [vaccinated versus non-vaccinated] for you to prevent flu infection?”) showed lower levels of negative health beliefs (i.e., barriers; standardized estimate = −0.221, *p* < 0.001). However, they were less likely to have had the flu vaccine than their counterparts (standardized estimate = −0.456, *p* < 0.001; Sobel test score = 4.03, *p* < 0.001). The model 2 finding indicates that although flu vaccine literacy significantly reduced ‘barrier’ belief, a higher level of the ‘barrier’ belief still significantly impeded flu vaccination behavior. Furthermore, the direct effect of flu vaccine literacy on flu vaccination was weakened through the ‘barrier’ belief.

Direct Effects. The ‘severity’ belief regarding flu infection, indicating the perception that flu can cause severe health problems, was not significantly impacted by flu vaccine literacy. Moreover, the ‘susceptibility’ belief indicating perceived vulnerability of individuals was not significantly influenced by flu vaccine literacy. Instead, flu vaccine literacy directly enhanced individuals’ vaccination behavior without mediation effects of health beliefs. As models 1–4 show (Figure 2), flu vaccine literacy significantly influenced flu vaccination (model 1 standardized estimate = 0.12, *p* = 0.017; model 3 standardized estimate = 0.154, *p* = 0.002; model 4 standardized estimate = 0.149, *p* = 0.003). However, flu vaccine literacy and flu vaccination did not significantly influence perceived physical health status, while the ‘susceptibility’ belief directly impacted perceived physical health status.

## 4. Discussions

The current research highlights the significance of flu shots among young adults, particularly under/graduate students, during the COVID-19 crisis [3]. This study highlighted the importance of the adherence of younger groups (under/graduate students) to immunization systems (i.e., flu vaccination). Its goal was to assess whether literacy about flu vaccines affects health attitudes, flu vaccination rates, and overall perceived health.

### 4.1. Vaccine Literacy, Health Beliefs, and Flu Vaccination

The present study confirms previous findings that flu vaccine literacy has a direct impact on flu vaccination (Figure 2: models 1, 3, 4) [19,46,47,48] and that individuals with a better understanding of vaccination tend to apply that understanding to their immunization behaviors [49]. The findings suggest that higher levels of vaccine literacy improve protective behaviors since they determine an individual’s ability to make informed decisions and access healthcare systems [50]. These results highlight the critical role that improving vaccine literacy plays in reducing flu infections, especially during pandemics. Furthermore, the study found that flu vaccine literacy affects health beliefs, such as the perceived benefits and severity of the flu, which in turn influence flu vaccination rates. Enhancing flu vaccine literacy can decrease negative attitudes and increase vaccination uptake, especially during pandemics.

Previous research found that health literacy significantly impacts health attitudes [51,52], and a relationship between ‘barrier beliefs’ and ‘benefit beliefs’ is known to affect vaccination rates [21,53]. Combining the Health Belief Model (HBM) [34] and the Health Literacy and Social Framework (HLSF) [35], this study supported the theoretical framework of the mediation effect of health beliefs on the relationship between health literacy and vaccination. A study by Niu et al. (2022) [53] in China discovered a mediation effect of ‘benefit belief’ between health literacy and preventive behavior. Biasio (2017) [50] emphasized the crucial role of the effective communication and monitoring of knowledge in increasing vaccine behavior, through empowerment that encompasses health literacy and health attitudes. The concept of ‘empowerment’ includes the role of health literacy and health beliefs and highlights the mediation role of health beliefs.

Notably, even though the belief in barriers acted as a mediator between vaccine literacy and flu vaccination, a higher level of barrier beliefs significantly hindered flu vaccination. This might be due to several reasons. First, the target group of this study was undergraduate and graduate students, so the group might be familiar with online information. It is also possible that a lower level of vaccine literacy, which may come from inaccurate information, could lead to vaccine hesitancy in these individuals [50]. Second, the form of vaccine literacy measure used in this study may also explain the significant impact of barrier beliefs on flu vaccination. The study asked, “Do you feel sure about the best choice for you to prevent flu infection (vaccinated or non-vaccinated) based on the given information?” This single-dimensional measure of vaccine literacy may not fully reflect the complex nature of the construct. Although the study used a validated measure of vaccine literacy that originated from the Ottawa Decision Support Framework [36], some limitations may exist in capturing the full definition of vaccine literacy using a single-dimensional approach. Researchers have noted that using a multidimensional approach, which takes into account various factors that affect health beliefs, may provide a more comprehensive understanding of an individual’s vaccine literacy [7,21,54]. This is because vaccine literacy is not simply knowledge about vaccines but a complex concept that requires a multidimensional approach to its study [50]. Last, family environment and parental influence can also contribute to the observed reluctance to receive flu vaccinations among undergraduate and graduate students. The collected data included a question about the influence of family on decisions to be vaccinated (response options: “not at all” to “very much”), and the results showed that the majority (80.6%) of the target group were influenced by family (parents, siblings, and other relatives). Previous research by Veldsijk et al. (2015) [55] found that parents with higher levels of vaccine hesitancy tended to have higher education levels and were less likely to be vaccinated. It was suggested that highly educated parents may be more skeptical and critical of their children’s vaccination, leading to higher vaccine hesitancy and decreased vaccination rates [56,57]. The study’s target population of undergraduate/graduate students was largely composed of highly educated individuals, with the majority of mothers (79.1%) and fathers (77.2%) having more than a college level of education. However, it is important to note that the relationship between educational attainment and vaccination behavior is complex and seldom straightforward. Some studies have found that lower educational attainment is associated with lower vaccination rates [46,58,59], while others have found the opposite. This study’s findings suggest that students may not be particularly concerned about the flu or related diseases, as the severity and susceptibility beliefs were not found to have a direct effect on flu vaccination rates.

### 4.2. Health Beliefs and Perceived Health Status

Higher perceived susceptibility to illness is associated with worse reported physical health, as previous studies have shown [4,60]. It may be related to individuals’ perceived vulnerabilities in healthcare. Multifaceted mechanisms of socioeconomic conditions can impact perceived vulnerability associated with an individual’s physical health status [61,62]. The direct impact of such vulnerability in healthcare on an individual’s perceived physical health is stronger than the impact of their use of healthcare services [60].

The results of this study show no significant relationship between flu vaccination, which is impacted by vaccine literacy and health beliefs, and perceived health status. This might be because undergraduate students hold a nonchalant attitude toward the flu and flu-related illnesses, viewing them as minor and not worth serious concern. They might also believe that the illnesses would not significantly affect their physical health and that they would recover quickly if they did get sick. In the same view, the current study finding indicated that ‘severity beliefs’ did not significantly influence flu vaccination, even perceived health status. This means that even if undergraduate or graduate students are aware of the severity of a flu infection, they may believe that they can recover quickly and that it will not have a significant long-term impact on their physical health status.

### 4.3. Implications

Healthcare providers or other practitioners in healthcare systems should establish and disseminate more effective education programs to enhance vaccine literacy. The concept of vaccine literacy is complex, and it has a significant impact on the health beliefs of undergraduate and postgraduate students. To address this issue effectively, education programs employing multicomponent strategies should be established; the multicomponent strategies should aim to reduce anti-vaccination perceptions, increase confidence in vaccination, enhance motivation to be vaccinated, and remove any perceived barriers that may prevent students from being vaccinated [50,63].

Education programs need to be customized for different age groups, particularly young and older adults. Undergraduate students should have access to information from trustworthy professionals who can provide accurate information about flu viruses and vaccinations. This will help them to understand the importance of vaccination and its impact on public health. Furthermore, governments should make mandatory educational opportunities accessible to young adults.

Governments should also establish official communication channels for older adults, such as parents, to dispel any doubts about the effects of vaccination. Clear and comprehensive communication can boost vaccination rates among younger generations, as they can be influenced by their family members. A prior study [64] recommended promoting health literacy by providing effective educational programs for both parents and younger groups, starting from primary and secondary schools.

Healthcare providers can provide accurate information to those who are hesitant to receive flu vaccines due to their beliefs rooted in biases or unreliable information [60]. Healthcare authorities should be aware of the factors related to vaccine literacy and the public’s vaccine literacy levels. Improving vaccine literacy and public health requires cooperation and collaboration between the public, policymakers, and healthcare providers, as the state of public health has far-reaching effects on social, economic, and environmental conditions.

Hiring competent healthcare providers, such as medical social workers or school counselors, can help promote vaccine adherence among undergraduate students. Community practitioners or representatives from organizations/institutions could visit colleges/universities to encourage students to be vaccinated. Additionally, professionals can educate not only students but also university professors and administrators about vaccines and their vital place in protecting public health [60].

### 4.4. Limitations

This study has several limitations. The sample group consisted of under/graduate students in Ohio, U.S., and the results may not reflect all U.S. under/graduate students. Using convenient sampling may limit the generalizability of the findings compared to random sampling, and a single dimension measure of vaccine literacy may not fully capture its complexity. The study found that most of the target groups had insurance coverage, which could be related to their health behavior. Since universities and colleges often offer free flu vaccines to their students, insurance status may not have a major effect on flu vaccination behavior. However, future studies should consider including a more diverse range of insurance statuses in the target group for greater generalizability. Future studies should consider using a multidimensional measure of vaccine literacy and design clinical trials with multifaceted approaches to improve vaccine literacy among young adults, including graduate and undergraduate students. In addition, some parts of the hypotheses of this study could not be confirmed: the direct or indirect impacts of literacy, beliefs, and flu vaccination on perceived health status were not significant. Despite the authors presenting several potential explanations for the insignificant effects of literacy, health beliefs, and flu vaccination on perceived health status, future studies may consider alternative methods for assessing perceived health status. Alternatively, future research could explore more specific health outcomes, such as experiences with hospitalization, flu-related infectious diseases, or immunity status.

## 5. Conclusions

This study investigated whether literacy about flu vaccines affects health attitudes, flu vaccination rates, and overall perceived health. Findings indicated that flu vaccine literacy directly influenced flu vaccination and that individuals with a better understanding of vaccination were more likely to adhere to immunization behaviors (e.g., vaccination). Importantly, this study found that flu vaccine literacy affected health beliefs (i.e., perceived benefits and severity of the flu) and then influenced flu vaccination rates. However, even though the belief in barriers acted as a mediator between vaccine literacy and flu vaccination, a higher level of barrier beliefs significantly impeded flu vaccination. Concerning the fact that vaccine literacy is not a simple construct, multicomponent strategies that can reduce ‘barrier beliefs’ should be established. Individuals receiving accurate/appropriate information (based on the multicomponent strategies) will eventually think that vaccination can positively influence their physical health.

## Figures and Tables

**Table 1:** Measure Details of Health Beliefs.

**Items**		**Response Option**
(1)	“I do not want to be vaccinated” (i.e., with the flu vaccine)	Participants were asked to rate their agreement with each item on a 5-point scale ranging from “strongly disagree” (1)~“strongly agree” (5).
(2)	“Flu vaccination has unpleasant side-effects”	
(3)	“In general I am opposed to vaccinations”	
(4)	“It is too much trouble for me to go to the doctor to be vaccinated”	
(5)	“Flu vaccinations weaken the natural immune system”	
(6)	“Flu vaccinations are too expensive”	
(7)	“Flu vaccinations are an effective protection against the flu”	
(8)	“I have an increased risk of falling ill with flu”	
(9)	“I am concerned about the risk of falling seriously ill”	
(10)	“I get sick more easily than other people my age”	
(11)	“Flu infection may lead to serious health problems”	
(12)	“If I had the flu, I would not be able to manage daily activities”	
(13)	“I am afraid the flu will make me very sick”	
(14)	“I am very worried about catching the flu”	
(15)	“Whenever I get sick it seems to be serious”	
(16)	“I cannot stand a flu infection because of my general health”	

Note: The 16 items were then grouped into four domains: Perceived Barriers (summing questions 1–6, with a Cronbach’s alpha of 0.794); Perceived Benefits (question 7, alpha score is not available);Perceived Susceptibility (summing questions 8–10, with a Cronbach’s alpha of 0.681);Perceived Severity (summing questions 11–16, with a Cronbach’s alpha of 0.771).

**Table 2:** Sample Characteristics.

Category	Response Options	*N* or *M*	% or *SD*
Flu vaccination	Yes	187	49.00
	No	195	51.00
Race	Whites	143	37.40
	African Americans	59	15.40
	Hispanics	69	18.10
	Asians	111	29.10
Gender	Male	100	26.2
	Female	282	73.8
Age		22.37	5.97
Family income (annual)	USD 0 to USD 44,999,	92	24.10
	USD 45,000 to USD 139,999	164	42.90
	above USD 140,000	126	33.00
Education (on average)	(0 = no formal education, 1 = 1st year, 2 = 2nd year, 3 = 3rd year, 4 = 4th year, 5 = 5th year, 6 = graduate school)	3.33 (on average, 3rd year)	1.60
Parent’s education (Mum; on average)	(0 = no formal education, 1 = elementary school, 2 = middle school, 3 = high school, 4 = some college, 5 = 2 years college, 6 = 4 years university, 7 = master’s degree, 8 = doctorate or higher)	5.45 (on average, 2 years college level)	1.69
Parent’s education (Dad; on average)	(0 = no formal education, 1 = elementary school, 2 = middle school, 3 = high school, 4 = some college, 5 = 2 years college, 6 = 4 years university, 7 = master’s degree, 8 = doctorate or higher)	5.45 (on average, 2 years college level)	2.02
Insurance	Insured	344	90.10
	Uninsured	38	9.90
Health Belief	Barrier (range: 6–30)	11.53	4.64
	Benefit (range: 1–5)	3.57	1.186
	Susceptibility (range: 3–15)	6.76	2.82
	Severity (range: 6–30)	16.42	4.73
Perceived Health Status	(range: 1–5)	3.71	0.88

Note: *N* = population number; *M* = mean score; *SD* = standardized deviation score.

## Data Availability

Utilized data were drawn from the survey (not open to the public to protect the survey participants).
